# Adjuvant Transarterial chemoembolization does not influence recurrence-free or overall survival in patients with combined hepatocellular carcinoma and Cholangiocarcinoma after curative resection: a propensity score matching analysis

**DOI:** 10.1186/s12885-020-07138-z

**Published:** 2020-07-10

**Authors:** Wei-Ren Liu, Meng-Xin Tian, Chen-Yang Tao, Zheng Tang, Yu-Fu Zhou, Shu-Shu Song, Xi-Fei Jiang, Han Wang, Pei-Yun Zhou, Wei-Feng Qu, Yuan Fang, Zhen-Bin Ding, Jian Zhou, Jia Fan, Ying-Hong Shi

**Affiliations:** 1 Department of Liver Surgery and Transplantation, Liver Cancer Institute, Zhongshan Hospital, Fudan University, 180 FengLin Road, Shanghai, 200032 China; 2 Key Laboratory of Carcinogenesis and Cancer Invasion of Ministry of Education, Shanghai, China; 3 Institutes of Biomedical Sciences, Fudan University, Shanghai, China; 4 Shanghai Key Laboratory of Organ Transplantation, Shanghai, China; 5 State Key Laboratory of Genetic Engineering and Collaborative Innovation Center for Genetics and Development, School of Life Sciences, Fudan University, Shanghai, China

**Keywords:** Combined hepatocellular carcinoma and intrahepatic cholangiocarcinoma, Transarterial chemoembolization, Overall survival, Disease-free survival, Propensity score matching analysis

## Abstract

**Background:**

The prognosis of patients with combined hepatocellular carcinoma and intrahepatic cholangiocarcinoma (CHC) is usually poor, and effective adjuvant therapy is missing making it important to investigate whether these patients may benefit from adjuvant transarterial chemoembolization (TACE). We aimed to evaluate the efficiency of adjuvant TACE for long-term recurrence and survival after curative resection before and after propensity score matching (PSM) analysis.

**Methods:**

In this retrospective study, of 230 patients who underwent resection for CHC between January 1994 and December 2014, 46 (18.0%) patients received adjuvant TACE. Univariate and multivariate regression analyses were used to identify the independent predictive factors of survival. Cox regression analyses and log-rank tests were used to compare overall survival (OS) and disease-free survival (DFS) between patients who did or did not receive adjuvant TACE.

**Results:**

A total of 230 patients (mean age 52.2 ± 11.9 years; 172 men) were enrolled, and 46 (mean age 52.7 ± 11.1 years; 38 men) patients received TACE. Before PSM, in multivariate regression analysis, γ-glutamyl transpeptidase (γ-GT), tumour nodularity, macrovascular invasion (MVI), lymphoid metastasis, and extrahepatic metastasis were associated with OS. Alanine aminotransferase (ALT), MVI, lymphoid metastasis, and preventive TACE (HR: 2.763, 95% CI: 1.769–4.314, *p* < 0.001) were independent prognostic factors for DFS. PSM created 46 pairs of patients. Before PSM, adjuvant preventive TACE was not associated with an increased risk of OS (HR: 0.911, 95% CI: 0.545–1.520, *p* = 0.720) or DFS (HR: 3.345, 95% CI: 1.686–6.638, *p* = 0.001). After PSM, the 5-year OS and DFS rates were comparable in the TACE group and the non-TACE group (OS: 22.7% vs 14.9%, respectively, *p* = 0.75; DFS: 11.2% vs 14.4%, respectively, *p* = 0.06).

**Conclusions:**

The present study identified that adjuvant preventive TACE did not influence DFS or OS after curative resection of CHC.

## Background

Primary liver cancer (PLC) is a heavy global health burden; it ranks as the second leading cause of mortality in men in less-developed countries, especially in China, which accounts for more than 50% of PLC patients in the world [1, 2]. PLC is composed of several biologically distinct subtypes: hepatocellular carcinoma (HCC), intrahepatic cholangiocarcinoma (ICC), and combined hepatocellular-cholangiocarcinoma (CHC). As a distinct and rare subtype of PLC, CHC accounts for less than 5% of PLC cases, with histological evidence of both hepatocellular and biliary epithelial differentiation [3, 4]. Due to the stem cell features of CHC, this disease is associated with an aggressive course and a poor prognosis, with 5-year overall survival (OS) ranging from 9.2–40% [5, 6].

Effective treatments for CHC are deficient. In our previous study, we found that radical surgical resection provided a better outcome that was intermediate between HCC and ICC [7, 8]. Aggressive surgical treatment, including lymph node dissection, may improve survival in patients diagnosed with CHC [9]. Regardless of Allen and Lisa class or the predominance of ICC cells within the tumour, the 5-year OS rate is 24% after hepatectomy [10]. Liver transplantation is not an appropriate therapeutic choice for CHC due to the disappointing results, with a mean OS of 11.7 months and a mean disease-free survival (DFS) of 7.97 months [11]. However, a group reported that very early CHC resulted in favourable post-transplant prognosis [12]. However, these studies had relatively small sample sizes and were retrospective in nature.

Similar to HCC and ICC, for CHC, recurrence is the most adverse factor influencing OS and DFS; vascular and lymph node invasion as well as the presence of satellite metastasis have been suggested as significant predictors of poor outcome after curative resection [13–15]. Transarterial chemoembolization (TACE), percutaneous ethanol injection (PEI) and radiofrequency ablation (RFA) are the most widely used treatments for HCC and post-resection recurrence [16–18]. For CHC, TACE shows an advantageous response and prognosis in recurrent patients after resection [19]. TACE is effective for prolonging the survival of patients with nonresectable CHC. Nonetheless, the effect of adjuvant TACE in CHC patients after curative resection is still unknown.

To address this issue, we conducted a retrospective cohort study to elucidate the relationship between adjuvant TACE and long-term recurrence and survival after curative resection of CHC using propensity score matching (PSM) and multivariate Cox regression analyses.

## Methods

### Participants and criteria

This was a retrospective study that used data collected at a single medical centre. The study was approved by the institutional review board and was in accordance with the standards of the Declaration of Helsinki and current ethical guidelines. Written informed consent was obtained for each patient. The inclusion and exclusion criteria are presented in the supplemental information.

**Fig. 1:**
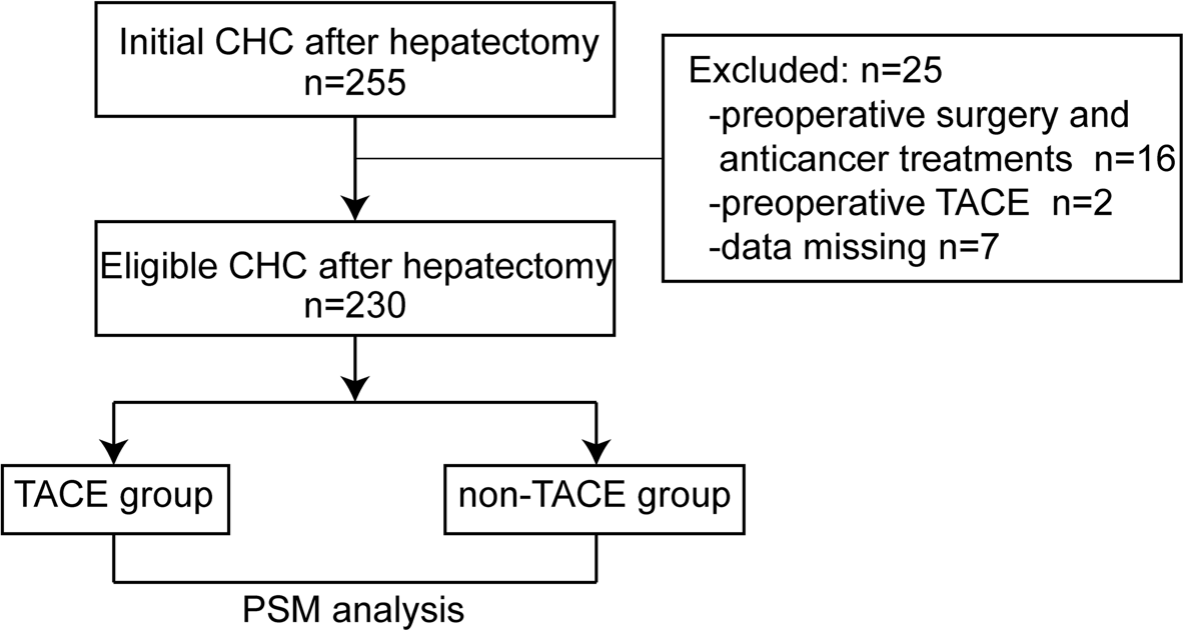
Patients selection flowchart

Between January 1994 and December 2014, a total of 255 patients who underwent curative hepatic resection and were diagnosed with CHC in the Department of Liver Surgery were retrospectively enrolled in this study. Among them, 25 patients who received preoperative surgery and anticancer treatments were excluded: 16 patients with a previous history of surgery, 2 patients who received preoperative TACE, and 7 patients with missing data. Thus, 230 patients were enrolled in the final analyses (Fig. 1). The detailed criteria for curative resection are shown in the supplemental information [20].

### TACE

The risk of recurrence after resection was assessed by tumour characteristics, which were established by the pathology report, and the patients with intermediate or high risks of recurrence were advised to undergo TACE therapy. A high risk of recurrence was defined as a single tumour with microvascular invasion or two or three tumours, and an intermediate risk of recurrence was defined as a solitary tumour larger than 5 cm without microvascular invasion [16, 21]. Using the Seldinger technique, a vascular catheter was inserted through a femoral artery to the hepatic artery, and hepatic angiography was then carried out. A microcatheter was used to inject Adriamycin (20–30 mg/m^2^) and lipiodol (3–5 mL) unselectively into the left and right hepatic arteries. The unselective embolization of the arterial tumor feeders was carried out by using 1-mm-diameter absorber gelatin sponge particles (Gelfoam; Upjohn, Kalamazoo, MI, USA) until arterial flow stasis was achieved.

### Follow-up

Patients were followed in our centre every 3 months until death or dropout (two patients) from the follow-up program. The median follow-up time was 15.1 months. The detailed follow-up procedures are shown in the supplemental information.

### Variables and outcomes

The data were prospectively collected and retrospectively reviewed. The detailed information from the database is shown in the supplemental information. The main outcomes of this study were OS and DFS. OS was measured from the date of the resection to either the date of death or the date of the last follow-up. DFS was defined from the date of the resection to the date of first recurrence or the date of death or the last follow-up visit.

### PSM

Patients in the TACE and non-TACE groups were matched using the PSM method [22], which was carried out using R software version 2.10.0 (R Project for Statistical Computing, https://www.r-project.org/, Austria). First, a propensity score (from 0 to 1) that contained the information of variates that was selected during matching was generated by logistic regression in PSM. Then, to create a reliable propensity score model, the variables that were chosen for matching included all the potential confounders [23, 24]. Thus, the variables contained all the independent prognostic factors of CHC. The Cox proportional hazards model was used to identify the independent prognostic factors, and the variables with statistical significance (*p* < 0.25) in univariate analysis were entered into multivariate analysis. The variables entered into the final propensity model were sex, ALT, perioperative blood transfusion, and lymphoid metastasis. Then, the model used one-to-one matching without replacement between TACE and non-TACE patients by using the nearest-neighbour matching algorithm. The calliper value was selected as 0.01, and the balance between the two groups after matching was evaluated by the standardized mean difference (*p* < 0.1).

### Statistical analysis

Statistical analyses were carried out using IBM SPSS 22.0 (SPSS Inc., Armonk, NY, USA) and SAS 9.1 (SAS Institute Inc., Cary, NC, USA). The demographic, clinical, and tumour characteristics were documented as summary statistics that were obtained using established methods. In both the TACE and non-TACE groups, continuous data were presented as the mean with a 25th–75th percentile range and analysed using Student’s *t* test or the Mann-Whitney *U* test. The categorical variables were presented as absolute and relative frequencies and compared by Pearson’s χ^2^ analysis or Fisher’s exact test. OS and DFS were compared using the Kaplan-Meier method, and survival differences between the two groups were analysed using the log-rank test. Multivariate Cox proportional hazard regression analyses were then carried out to adjust for other prognostic factors that were associated with OS and DFS. Moreover, to strengthen the accuracy of the model, a robust sandwich variance estimator was used in all the cohorts for estimating the hazard ratios and their 95% confidence intervals (CIs). All tests using two-tailed *p* < 0.05 were considered to be statistically significant.

## Results

### Demographic and clinicopathological characteristics

Table 1 summarizes the baseline characteristics of patients with CHC who underwent TACE (*n* = 46) and those who did not (*n* = 184) before PSM. The mean age of patients in the TACE group (52 ± 10.7 years) was similar to that of patients in the non-TACE group (52.3 ± 12.1 years), and the sex distribution was similar in both groups (38 and 134 male patients in the TACE group and non-TACE group, respectively). The median AFP (*p* = 0.006), median bilirubin (*p* < 0.001), occlusion time (*p* = 0.044), and macrovascular invasion (*p* = 0.041) were higher in the TACE group than in the non-TACE group, and the median CA19–9 was higher in the non-TACE group than in the TACE group (*p* = 0.029). After PSM, the mean age of patients in the TACE group (52 ± 10.7 years) was similar to that of patients in the non-TACE group (53.4 ± 11.6 years), and the sex distribution was similar in both groups. Except for the higher median AFP (*p* = 0.006), lower median CA19–9 (*p* = 0.023), lower median bilirubin (< 0.001), lower mean occlusion time (*p* = 0.044), and macrovascular invasion (*p* = 0.041) in the TACE group, there were no significant differences between the TACE group and the non-TACE group in terms of the baseline characteristics (*p* > 0.05).

**Table 1:** Preoperative clinicopathologic Data of Patients with CHC Who received or not postoperative TACE

Variable	Before Propensity Matching			After Propensity Matching		
	Without TACE (*n* = 184)	Postoperative TACE (*n* = 46)	*P*	Without TACE (*n* = 46)	Postoperative TACE (*n* = 46)	*P*
Sex			0.172			> 0.99
Men	134	38		38	38	
Women	50	8		8	8	
Mean age (y)	52.3 ± 12.1	52 ± 10.7	0.326	53.4 ± 11.6	52 ± 10.7	0.834
HBsAg			> 0.99			0.810
Positive	136	34		35	34	
Negative	48	12		11	12	
HBcAb			0.666			0.231
Positive	153	9		42	9	
Negative	31	37		4	37	
HCV antibody			> 0.99			> 0.99
Positive	4	1		1	1	
Negative	180	45		45	45	
Median AFP, ng/mL	24.7 (1–80,000)	96 (1.8–46,897)	**0.006**	21.3 (1–30,728)	96 (1.8–46,897)	**0.002**
Median CEA, μg/mL	2.5 (0–274)	2.1 (0.5–70.5)	0.364	2.7 (0.1–112.4)	2.1 (0.5–70.5)	0.423
Median CA19–9, U/ml	28.1 (0–4370)	19.4 (0.2–300.1)	**0.029**	22 (0.5–4062.5)	19.4 (0.2–300.1)	**0.023**
Median bilirubin, μmol/L	11.8 (1.7–314.8)	12.9 (5.7–156.5)	**< 0.001**	13.7 (2.4–169.3)	12.9 (5.7–156.5)	0.664
Median albumin, g/L	41 (26–55)	42 (35–66)	0.397	41 (30–48)	42 (35–66)	0.556
Median ALT, U/L	28 (5–484)	31 (5–104)	0.094	26 (11–484)	31 (5–104)	0.109
Median ALP, IU/L	89.5 (22–1413)	88.5 (46–184)	0.477	92 (25–331)	88.5 (46–184)	0.599
Median GGT, U/L	59 (3.6–1632)	80 (18–490)	0.923	75.5 (10–658)	80 (18–490)	0.273
Median platelets, 10^3^/μL	13.7 (2.2–47.6)	16 (3.9–46.1)	0.319	15.3 (5.3–24.7)	16 (3.9–46.1)	0.171
Median prothrombin time, s	11.8 (9–17.6)	12 (10.2–13.8)	0.941	12 (10.2–14.6)	12 (10.2–13.8)	0.903
Median INR	1 (0.5–1.5)	1 (0.8–1.2)	0.227	1 (0.5–1.2)	1 (0.8–1.2)	0.065
Median tumour size, cm	5 (1–24)	7.3 (1.5–17)	0.626	6 (1.5–22)	7.3 (1.5–17)	0.384
Median tumour nodularities	1 (1–10)	1 (1–5)	0.140	1 (1–6)	1 (1–5)	0.648
Median blood loss, ml	200 (30–3500)	200 (10–2500)	0.182	200 (50–1800)	200 (10–2500)	0.480
Mean occlusion, min	6.8 ± 8.6	10 ± 1.6	**0.044**	5.4 ± 1.1	10 ± 1.6	0.090
Macrovascular invasion			**0.041**			> 0.99
Positive	11	7		7	7	
Negative	173	39		39	39	
Microvascular invasion			0.689			0.607
Positive	39	11		8	11	
Negative	145	35		38	35	
Lymphoid metastasis			0.840			> 0.99
Positive	22	6		6	6	
Negative	162	40		40	40	
Extrahepatic metastasis			0.719			0.646
Positive	6	2		3	2	
Negative	178	44		43	44	
Postrecurrent therapy			0.451			0.583
Resection	2	1		1	1	
TACE	27	6		10	6	
Regional therapy	4	1		1	1	
Chemothearpy	66	14		11	14	
Selective internal radiation therapy	5	2		1	2	
Stereotactic body radiation	12	5		3	5	
Best supportive care	58	17		19	17	

Data are numbers of patients. Data in parentheses are range. Mean data are±standard deviation. Regional therapy: Radiofrequency ablation and percutaneous ethanol injection

*HBsAg* hepatitis B surface antigen, *HBcAb* hepatitis B core antibody, *HCV* hepatitis C virus, *AFP* α-fetoprotein, *CEA* carcino-embryonic antigen, *CA19–9* carbohydrate 19–9, *INR* International normalized ratio, *ALT* alanine aminotransferase, *GGT* γ-glutamyl transpeptidase, *ALP* alkaline phosphatase, *MVI* microvascular vascular invasion

### OS and DFS before PSM

**Fig. 2:**
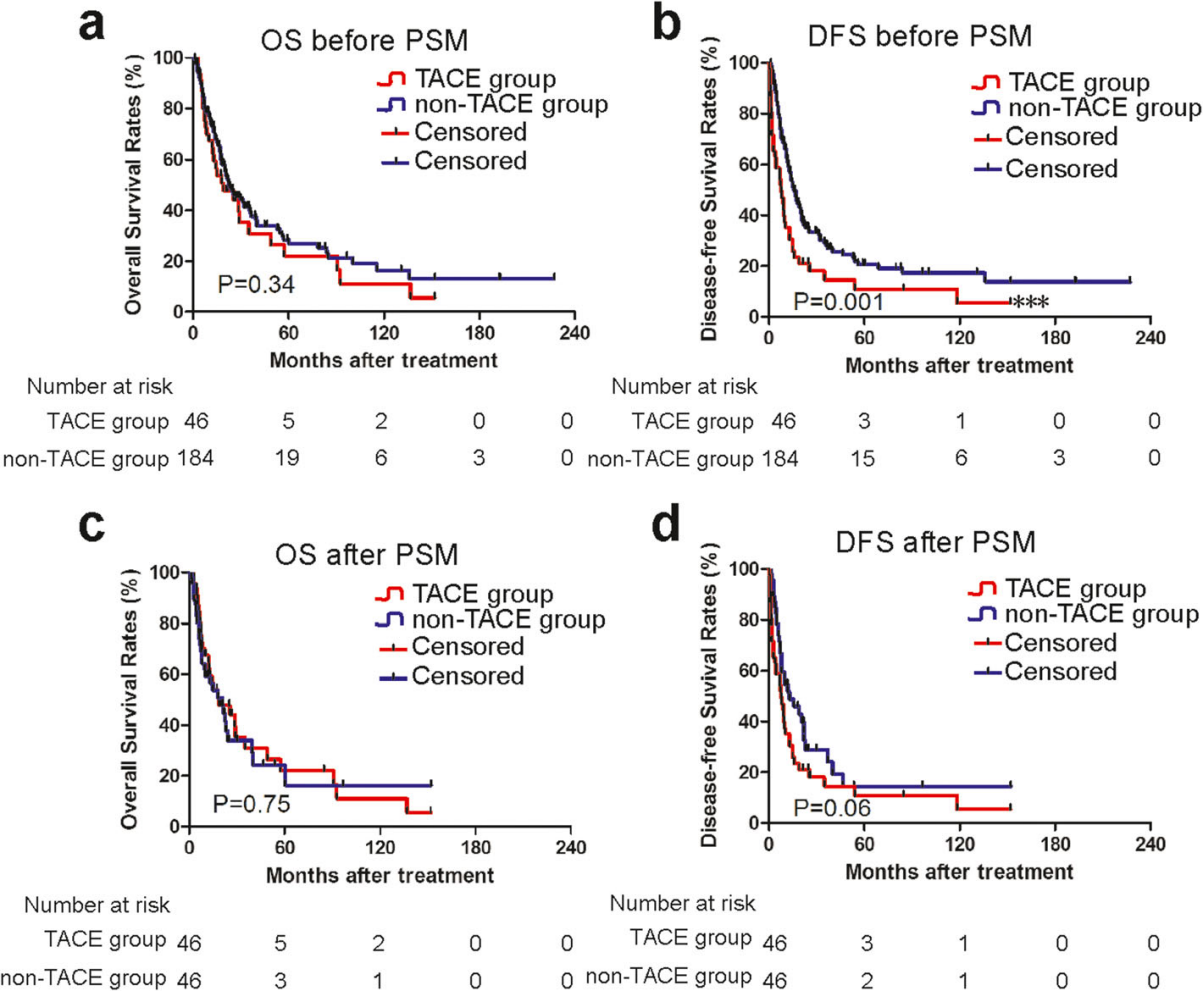
Kaplan-Meier curves of survival outcomes of adjuvant TACE in patients with CHC before and after PSM analysis. Kaplan-Meier curves of (**a**) overall survival (OS) and (**b**) disease-free survival (DFS) for patients with CHC before propensity score matching analysis; Kaplan-Meier curves of (**c**) overall survival (OS) and (**d**) disease-free survival (DFS) for patients with CHC after PSM analysis

The median survival of the whole cohort was 22.6 months, and the overall cumulative OS rates at 1, 3, 5, and 10 years were 48.5, 33.3, 25.8, and 15.3%, respectively. The median OS of the TACE group and non-TACE group was 22.0 months and 23.5 months, respectively. The cumulative OS rates were comparable between the two groups; the 1-, 3-, 5-, and 10-year OS rates in the TACE group were 46.6, 31.7, 22.7, and 12.6%, respectively, whereas those in the non-TACE group were 49.0, 33.7, 26.6, and 16.1%, respectively (*p* = 0.34) (Fig. 2a). The median DFS of the whole cohort was 14.0 months, and the cumulative DFS rates at 1, 3, 5, and 10 years were 20.9, 10.4, 0.7, and 0.3%, respectively. Stratified by TACE, the median DFS in the TACE group was less than that in the non-TACE group (9.3 months vs. 17.2 months) (*p* = 0.001) (Fig. 2b).

### The prognostic factors of CHC before PSM

To identify potential confounders, we used the Cox proportional hazards model to analyse the risk factors for CHC. For OS, in univariate analysis, the following six variants were enrolled in the multivariate analysis: γ-GT (*p* < 0.001), tumour size (*p* = 0.002), tumour nodularities (*p* = 0.003), macrovascular invasion (*p* < 0.001), lymphoid metastasis (*p* < 0.001), and extrahepatic metastasis (*p* < 0.001). In multivariate analysis, γ-GT (*p* = 0.001), tumour nodularities (*p* = 0.031), macrovascular invasion (*p* < 0.001), lymphoid metastasis (*p* = 0.008), and extrahepatic metastasis (*p* < 0.001) were independent factors of OS (Table 2).

**Table 2:** Univariable and multivariable cox analysis of OS before propensity matched analysis

Variable	Univariable			Multivariable		
	HR	95% CI	*P*	HR	95% CI	*P*
Age (≥60/< 60, year)	1.279	0.857–1.908	0.229	–	–	–
Sex (Men/Women)	1.443	0.95–2.193	0.085	–	–	–
HBsAg (yes/no)	1.044	0.719–1.517	0.821	–	–	–
HCV antibody (yes/no)	2.293	0.722–7.283	0.159	–	–	–
AFP (≥20/< 20, ng/mL)	2.819	0.68–11.682	0.153	–	–	–
CEA (≥5/<5, ng/mL)	1.844	0.643–5.29	0.255	–	–	–
CA19–9 (≥37/<37, U/mL)	2.069	0.639–6.702	0.225	–	–	–
Liver cirrhosis, yes (%)	1.252	0.857–1.83	0.245	–	–	–
TB (≥17/< 17, μmol/L)	0.950	0.626–1.443	0.810	–	–	–
ALB (≥40/<40, g/mL)	0.759	0.530–1.086	0.132	–	–	–
ALT (≥35/<35, U/L)	1.327	0.941–1.870	0.106	–	–	–
γ-GT (≥40/<40, U/L)	2.662	1.703–4.163	**< 0.001**	2.152	1.354–3.421	**0.001**
PLT (≥10/< 10 10^3^/μL)	1.005	0.665–1.518	0.982	–	–	–
Prothrombin time, median (range), s	1.199	0.781–1.841	0.406	–	–	–
Tumour size, cm	1.769	1.235–2.534	**0.002**	1.274	0.867–1.872	0.218
Tumour nodularities	1.167	1.055–1.292	**0.003**	1.130	1.011–1.262	**0.031**
Occlusion, min (< 20/≥20)	0.290	0.740–2.250	0.369	–	–	–
Macrovascular invasion (yes/no)	1.927	1.442–2.576	**< 0.001**	1.869	1.375–2.540	**< 0.001**
Microvascular invasion (yes/no)	1.365	0.921–2.204	0.122	–	–	–
Lymphoid metastasis (yes/no)	2.801	1.745–4.495	**< 0.001**	2.031	1.201–3.435	**0.008**
Extrahepatic metastasis (yes/no)	11.435	5.262–24.849	**< 0.001**	6.392	2.731–14.961	**< 0.001**
Preventive TACE (yes/no)	1.212	0.807–1.821	0.354	–	–	–

*HBsAg* hepatitis B surface antigen, *HCV* hepatitis C virus, *AFP* α-fetoprotein, *CEA* carcino-embryonic antigen, *CA19–9* carbohydrate 19–9, *TB* total bilirubin, *ALB* albumin, *ALT* alanine aminotransferase, *γ-GT* γ-glutamyl transpeptidase, *PLT* platelet, *ALP* alkaline phosphatase

For DFS, in univariate analysis, the following five variants were enrolled in the multivariate analysis: male sex (*p* = 0.034), ALT (*p* = 0.008), γ-GT (*p* = 0.016), occlusion time (*p* = 0.002), macrovascular invasion (*p* = 0.001), lymphoid metastasis (*p* = 0.005), and preventive TACE (*p* < 0.001). In multivariate analysis, we found that ALT (*p* = 0.031), macrovascular invasion (*p* = 0.001), lymphoid metastasis (*p* = 0.001), and preventive TACE (HR: 2.763, 95% CI: 1.769–4.314, *p* < 0.001) were independent prognostic factors of DFS (Table 3).

**Table 3:** Univariable and multivariable cox analysis of DFS before propensity matched analysis

Variable	Univariable			Multivariable		
	HR	95% CI	*P*	HR	95% CI	*P*
Age (≥60/< 60, year)	1.240	0.765–2.010	0.382	–	–	–
Sex (Men/Women)	1.751	1.042–2.941	**0.034**	1.919	1.097–3.357	**0.022**
HBsAg (yes/no)	0.672	0.405–1.114	0.123	–	–	–
HCV antibody (yes/no)	0.782	0.108–5.636	0.807	–	–	–
AFP (≥20/< 20, ng/mL)	1.245	0.824–1.881	0.299	–	–	–
CEA (≥5/<5, ng/mL)	1.169	0.672–2.035	0.581	–	–	–
CA19–9 (≥37/<37, U/mL)	1.136	0.727–1.775	0.575	–	–	–
Liver cirrhosis, yes (%)	1.291	0.815–2.044	0.277	–	–	–
TB (≥17/< 17, μmol/L)	0.998	0.607–1.641	0.995	–	–	–
ALB (≥40/<40, g/mL)	0.771	0.499–1.191	0.241	–	–	–
ALT (≥35/<35, U/L)	1.741	1.154–2.267	**0.008**	1.676	1.050–2.677	**0.031**
γ-GT (≥40/<40, U/L)	1.811	1.116–2.938	**0.016**	1.105	0.653–1.870	0.711
PLT (≥10/< 10 10^3^/μL)	0.856	0.529–1.382	0.524	–	–	–
Prothrombin time, median (range), s	1.417	0.845–2.375	0.186	–	–	–
Tumour size, cm	1.226	0.809–1.857	0.338	–	–	–
Tumour nodularities	1.056	0.918–1.215	0.442	–	–	–
Occlusion, min (< 20/≥20)	2.363	1.356–4.119	**0.002**	1.790	0.974–3.289	0.061
Macrovascular invasion (yes/no)	1.878	1.300–2.713	**0.001**	2.026	1.342–3.058	**0.001**
Microvascular invasion (yes/no)	1.084	0.654–1.797	0.754	–	–	–
Lymphoid metastasis (yes/no)	2.300	1.287–4.112	**0.005**	2.835	1.517–5.297	**0.001**
Extrahepatic metastasis (yes/no)	2.248	0.538–9.395	0.267	–	–	–
Preventive TACE (yes/no)	2.799	1.815–4.317	**< 0.001**	2.763	1.769–4.314	**< 0.001**

*HBsAg* hepatitis B surface antigen, *HCV* hepatitis C virus, *AFP* α-fetoprotein, *CEA* carcino-embryonic antigen, *CA19–9* carbohydrate 19–9, *TB* total bilirubin, *ALB* albumin, *ALT* alanine aminotransferase, *γ-GT* γ-glutamyl transpeptidase, *PLT* platelet, *ALP* alkaline phosphatase

### PSM for TACE and non-TACE patients

The distribution of the risk factors and demographic characteristics differed between the TACE and non-TACE groups. To reduce confounding factors and to reflect the true effect of TACE, we established a PSM model based on the analysis of the risk factors described above. Considering OS and DFS, four variates were involved in the model: AFP, CA19–9, total bilirubin, and macrovascular invasion. Finally, we matched 46 pairs of TACE and non-TACE patients. Apart from AFP and CA19–9, all other variables were balanced between the two groups (all *p* > 0.2). The balances between the two groups are shown in Table 1.

### OS and DFS after PSM

After PSM, the median OS of the TACE group and non-TACE group was 22.0 months and 16.3 months, respectively. The cumulative survival rates in the TACE group at 1, 3, 5, and 10 years were 46.6, 31.7, 22.7, and 12.6%, respectively, whereas those in the non-TACE group were 36.4, 22.4, 14.9, and 14.9%, respectively. However, the OS between the TACE and non-TACE groups was still comparable after PSM (*p* = 0.75) (Fig. 2c). The median DFS of the TACE group and non-TACE group was 7.3 months and 10.0 months, respectively. The cumulative DFS rates in the TACE group at 1, 3, 5, and 10 years were 20.8, 14.9, 11.2, and 5.6%, respectively, whereas those in the non-TACE group were 28.7, 14.4, 14.4, and 14.4%, respectively. However, the DFS between the TACE and non-TACE groups was comparable after PSM (*p* = 0.06) (Fig. 2d).

### The prognostic factors of CHC after PSM

After PSM, for OS, in univariate analysis, the following three variants were enrolled in the multivariate analysis: HCV antibody (*p* = 0.013), macrovascular invasion (*p* < 0.001), and extrahepatic metastasis (*p* < 0.001). In multivariate analysis, HCV antibody (*p* = 0.004), macrovascular invasion (*p* = 0.001), and extrahepatic metastasis (*p* < 0.001) were independent factors of OS (Table 4).

**Table 4:** Univariable and multivariable cox analysis of OS after propensity matched analysis

Variable	Univariable			Univariable		
	HR	95% CI	*P*	HR	95% CI	*P*
Age (≥60/< 60, year)	0.922	0.463–1.837	0.818	–	–	–
Sex (Men/Women)	1.458	0.689–3.087	0.324	–	–	–
HBsAg (yes/no)	1.711	0.887–3.300	0.109	–	–	–
HCV antibody (yes/no)	6.405	1.491–27.524	**0.013**	9.142	2.028–41.225	**0.004**
AFP (≥20/< 20, ng/mL)	1.288	0.761–2.181	0.346	–	–	–
CEA (≥5/<5, ng/mL)	1.643	0.924–2.923	0.091	–	–	–
CA19–9 (≥37/<37, U/mL)	1.591	0.932–2.715	0.089	–	–	–
Liver cirrhosis, yes (%)	1.952	1.091–3.493	**1.379**	6.264	0.734–2.590	0.318
TB (≥17/< 17, μmol/L)	0.739	0.383–1.427	0.368	–	–	–
ALB (≥40/<40, g/mL)	0.814	0.476–1.391	0.451	–	–	–
ALT (≥35/<35, U/L)	1.459	0.869–2.452	0.153	–	–	–
γ-GT (≥40/<40, U/L)	1.811	0.933–3.515	0.079	–	–	–
PLT (≥10/< 10 10^3^/μL)	1.353	0.683–2.682	0.386	–	–	–
Prothrombin time, median (range), s	1.014	0.547–1.880	0.964	–	–	–
Tumour size, cm	1.466	0.814–2.639	0.203	–	–	–
Tumour nodularities	1.017	0.785–1.318	0.898	–	–	–
Occlusion, min (< 20/≥20)	1.560	0.735–3.310	0.247	–	–	–
Macrovascular invasion (yes/no)	3.343	1.770–6.315	**< 0.001**	3.035	1.543–5.972	**0.001**
Microvascular invasion (yes/no)	1.359	0.725–2.546	0.338	–	–	–
Lymphoid metastasis (yes/no)	1.487	0.667–3.315	0.332	–	–	–
Extrahepatic metastasis (yes/no)	6.805	2.549–18.166	**< 0.001**	6.264	2.277–17.235	**< 0.001**
Preventive TACE (yes/no)	0.911	0.545–1.520	0.720	–	–	–

*HBsAg* hepatitis B surface antigen, *HCV* hepatitis C virus, *AFP* α-fetoprotein, *CEA* carcino-embryonic antigen, *CA19–9* carbohydrate 19–9, *TB* total bilirubin, *ALB* albumin, *ALT* alanine aminotransferase, *γ-GT* γ-glutamyl transpeptidase, *PLT* platelet, *ALP* alkaline phosphatase

For DFS, in univariate analysis, the following four variants were enrolled in the multivariate analysis: ALT (*p* = 0.02), occlusion time (*p* = 0.005), macrovascular invasion (*p* = 0.002), and preventive TACE (*p* = 0.001). In multivariate analysis, macrovascular invasion (*p* = 0.006) and preventive TACE (HR: 3.345, 95% CI: 1.686–6.638, *p* = 0.001) were independent factors of DFS (Table 5).

**Table 5:** Univariable and multivariable cox analysis of DFS after propensity matched analysis

Variable	Univariable			Multivariable		
	HR	95% CI	*P*	HR	95% CI	*P*
Age (≥60/< 60, year)	1.198	0.587–2.443	0.620	–	–	–
Sex (Men/Women)	1.827	0.713–4.685	0.209	–	–	–
HBsAg (yes/no)	1.478	0.706–3.096	0.300	–	–	–
HCV antibody (yes/no)	0.048	0.526–4.934	0.665	–	–	–
AFP (≥20/< 20, ng/mL)	1.075	0.585–1.976	0.815	–	–	–
CEA (≥5/<5, ng/mL)	0.820	0.380–1.771	0.614	–	–	–
CA19–9 (≥37/<37, U/mL)	1.019	0.520–1.997	0.957	–	–	–
Liver cirrhosis, yes (%)	1.436	0.752–2.744	0.273	–	–	–
TB (≥17/< 17, μmol/L)	0.941	0.449–1.973	0.873	–	–	–
ALB (≥40/<40, g/mL)	0.580	0.315–1.068	0.080	–	–	–
ALT (≥35/<35, U/L)	2.083	1.120–3.873	**0.020**	1.989	0.980–4.037	0.057
γ-GT (≥40/<40, U/L)	1.265	0.616–2.597	0.521	–	–	–
PLT (≥10/< 10 10^3^/μL)	0.975	0.466–2.043	0.947	–	–	–
Prothrombin time, median (range), s	1.841	0.942–3.598	0.074	–	–	–
Tumour size, cm	1.077	0.560–2.071	0.823	–	–	–
Tumour nodularities	0.992	0.731–1.346	0.957	–	–	–
Occlusion, min (< 20/≥20)	3.308	1.388–6.647	**0.005**	1.565	0.639–3.833	0.327
Macrovascular invasion (yes/no)	3.703	1.607–8.535	**0.002**	3.361	1.416–7.977	**0.006**
Microvascular invasion (yes/no)	1.705	0.854–3.407	0.131	–	–	–
Lymphoid metastasis (yes/no)	1.423	0.553–3.663	0.464	–	–	–
Extrahepatic metastasis (yes/no)	2.246	0.520–9.712	0.279	–	–	–
Preventive TACE (yes/no)	3.144	1.610–6.137	**0.001**	3.345	1.686–6.638	**0.001**

*HBsAg* hepatitis B surface antigen, *HCV* hepatitis C virus, *AFP* α-fetoprotein, *CEA* carcino-embryonic antigen, *CA19–9* carbohydrate 19–9, *TB* total bilirubin, *ALB* albumin, *ALT* alanine aminotransferase, *γ-GT* γ-glutamyl transpeptidase, *PLT* platelet, *ALP* alkaline phosphatase, *NS* non-sense

## Discussion

CHC is a rare and complex disease with limited treatment options. In our previous study, we constructed a convenient and reliable prediction model for identifying individuals with CHC. In this model, 2.73% of the patients diagnosed with liver cancer were definitely diagnosed with CHC [6]. However, even with curative resection, the prognosis of CHC is dismal. Due to its more malignant behaviour than HCC, CHC tends to recur after curative resection [13]. Herein, we answered this difficult question: can we prolong the survival of CHC patients after curative resection? We found that postoperative adjuvant TACE could not prolong DFS in CHC patients after curative resection.

Regarding HCC recurrence, many postoperative adjuvant therapies, including targeted therapy, have reported limited success [20, 25, 26]. In our previous retrospective study, postoperative adjuvant TACE prolonged the survival of patients with risk factors [27, 28]. In our prospective study, we found that adjuvant TACE significantly reduced tumour recurrence and improved RFS and OS in patients with HBV-related HCC who had an intermediate or high risk for recurrence [16]. Regarding ICC recurrence, ICC patients with high nomogram scores benefited from adjuvant TACE following liver resection [29].

In CHC management, TACE is considered to be inefficient, as CHC has less vasculature and is much more fibrotic than HCC [30]. However, one study showed that TACE was effective for prolonging the survival of patients with nonresectable CHC, and the survival period after TACE was dependent on tumour size, tumour vascularity, liver function, and the presence or absence of portal vein invasion [31]. According to the enhanced pattern, the globally enhancing type showed a better response and prognosis after TACE than the peripherally enhancing type [19]. In our view, as CHC is less vascular and much more fibrotic than HCC, thus CHC is less likely to respond to TACE [30], which may contribute to the inefficiency of postoperative adjuvant TACE in CHC patients.

This study has several limitations. First, this is a retrospective cohort study but not a randomized controlled trial. The initial surgical approach in patients with CHC has changed over the last 20 years, as especially lymphadenectomy was not performed regularly in the early years, and approaches to CHC might have changed due to the CCC component as well. Thus, a randomized trial is warranted to reduce the bias of patients’ selection and so on. As was done in the present study, it is the best-suited study design to apply PSM and multivariate Cox regression analyses. Second, our study is based on a single institution, and external confirmation is urgently needed in our future work. Third, the HBV rate was higher than the rates published from Western countries, which may cause bias in clinical decision-making. Finally, we found that adjuvant TACE shortened DFS and did not affect OS in CHC patients, as OS and DFS were influenced by tumour characteristics and treatment modalities. Further, the individual decision on postrecurrence treatment would affect the prognosis of each patient. Thus, whether adjuvant TACE affects OS and DFS also needs further investigation.

## Conclusions

To summarize, with the use of propensity score analyses and multivariate Cox regression analyses, our present study showed that adjuvant TACE shortened DFS and did not affect OS in CHC patients. Our study showed that more specific criteria, such as tumour enhancement type, should be warranted for select patients who will benefit from postoperative adjuvant TACE.

## Supplementary information

**Additional file 1.**

## Data Availability

The datasets used and analyzed during the current study are available from the corresponding author on reasonable request.

## Abbreviations

AFP: α-fetoprotein
ALP: Alkaline phosphatase
ALT: Alanine aminotransferase
CA19–9: Carbohydrate 19–9
CEA: Carcino-embryonic antigen
CHC: Combined hepatocellular carcinoma and intrahepatic cholangiocarcinoma
CI: Confidence interval
DFS: Disease-free survival
γ-GT: γ-glutamyl transpeptidase
HBcAb: Hepatitis B core antibody
HBsAg: Hepatitis B surface antigen
HCV: Hepatitis C virus
HCC: Hepatocellular carcinoma
ICC: Intrahepatic cholangiocarcinoma
INR: International normalized ratio
MVI: Vascular invasion
OS: Overall survival
PEI: Percutaneous ethanol injection
PLC: Primary liver cancer
PSM: Propensity score matching
RFA: Radiofrequency ablation
TACE: Transarterial chemoembolization

## References

[1] Torre LA, Bray F, Siegel RL, Ferlay J, Lortet-Tieulent J, Jemal A (2015). Global cancer statistics, 2012. CA Cancer J Clin.

[2] Allemani C, Matsuda T, Di Carlo V, Harewood R, Matz M, Niksic M, Bonaventure A, Valkov M, Johnson CJ, Esteve J (2018). Global surveillance of trends in cancer survival 2000-14 (CONCORD-3): analysis of individual records for 37 513 025 patients diagnosed with one of 18 cancers from 322 population-based registries in 71 countries. Lancet.

[3] Garancini M, Goffredo P, Pagni F, Romano F, Roman S, Sosa JA, Giardini V (2014). Combined hepatocellular-cholangiocarcinoma: a population-level analysis of an uncommon primary liver tumor. Liver Transpl.

[4] Brunt E, Aishima S, Clavien PA, Fowler K, Goodman Z, Gores G, Gouw A, Kagen A, Klimstra D, Komuta M (2018). cHCC-CCA: consensus terminology for primary liver carcinomas with both hepatocytic and cholangiocytic differentation. Hepatology.

[5] Gera S, Ettel M, Acosta-Gonzalez G, Xu R (2017). Clinical features, histology, and histogenesis of combined hepatocellular-cholangiocarcinoma. World J Hepatol.

[6] Tian MX, He WJ, Liu WR, Yin JC, Jin L, Tang Z, Jiang XF, Wang H, Zhou PY, Tao CY (2018). A novel risk prediction model for patients with combined hepatocellular-Cholangiocarcinoma. J Cancer.

[7] Yin X, Zhang BH, Qiu SJ, Ren ZG, Zhou J, Chen XH, Zhou Y, Fan J (2012). Combined hepatocellular carcinoma and cholangiocarcinoma: clinical features, treatment modalities, and prognosis. Ann Surg Oncol.

[8] Tao CY, Liu WR, Jin L, Tang Z, Tian MX, Jiang XF, Wang H, Zhou PY, Fang Y, Ding ZB (2018). Surgical treatment of combined hepatocellular-Cholangiocarcinoma is as effective in elderly patients as it is in younger patients: a propensity score matching analysis. J Cancer.

[9] Kim KH, Lee SG, Park EH, Hwang S, Ahn CS, Moon DB, Ha TY, Song GW, Jung DH, Kim KM (2009). Surgical treatments and prognoses of patients with combined hepatocellular carcinoma and cholangiocarcinoma. Ann Surg Oncol.

[10] Ariizumi S, Kotera Y, Katagiri S, Nakano M, Yamamoto M (2012). Combined hepatocellular-cholangiocarcinoma had poor outcomes after hepatectomy regardless of Allen and Lisa class or the predominance of intrahepatic cholangiocarcinoma cells within the tumor. Ann Surg Oncol.

[11] Magistri P, Tarantino G, Serra V, Guidetti C, Ballarin R, Di Benedetto F (2017). Liver transplantation and combined hepatocellular-cholangiocarcinoma: feasibility and outcomes. Digest Liver Dis.

[12] Jung DH, Hwang S, Song GW, Ahn CS, Moon DB, Kim KH, Ha TY, Park GC, Hong SM, Kim WJ (2017). Longterm prognosis of combined hepatocellular carcinoma-cholangiocarcinoma following liver transplantation and resection. Liver Transpl.

[13] Koh KC, Lee H, Choi MS, Lee JH, Paik SW, Yoo BC, Rhee JC, Cho JW, Park CK, Kim HJ (2005). Clinicopathologic features and prognosis of combined hepatocellular cholangiocarcinoma. Am J Surg.

[14] Tang D, Nagano H, Nakamura M, Wada H, Marubashi S, Miyamoto A, Takeda Y, Umeshita K, Dono K, Monden M (2006). Clinical and pathological features of Allen's type C classification of resected combined hepatocellular and cholangiocarcinoma: a comparative study with hepatocellular carcinoma and cholangiocellular carcinoma. J Gastrointest Surg.

[15] Sanada Y, Shiozaki S, Aoki H, Takakura N, Yoshida K, Yamaguchi Y (2005). A clinical study of 11 cases of combined hepatocellular-cholangiocarcinoma assessment of enhancement patterns on dynamics computed tomography before resection. Hepatol Res.

[16] Wang Z, Ren Z, Chen Y, Hu J, Yang G, Yu L, Yang X, Huang A, Zhang X, Zhou S (2018). Adjuvant Transarterial chemoembolization for HBV-related hepatocellular carcinoma after resection: a randomized controlled study. Clin Cancer Res.

[17] Llovet JM, Real MI, Montana X, Planas R, Coll S, Aponte J, Ayuso C, Sala M, Muchart J, Sola R (2002). Arterial embolisation or chemoembolisation versus symptomatic treatment in patients with unresectable hepatocellular carcinoma: a randomised controlled trial. Lancet.

[18] Peng ZW, Zhang YJ, Chen MS, Xu L, Liang HH, Lin XJ, Guo RP, Zhang YQ, Lau WY (2013). Radiofrequency ablation with or without transcatheter arterial chemoembolization in the treatment of hepatocellular carcinoma: a prospective randomized trial. J Clin Oncol.

[19] Na SK, Choi GH, Lee HC, Shin YM, An J, Lee D, Shim JH, Kim KM, Lim YS, Chung YH (2018). The effectiveness of transarterial chemoembolization in recurrent hepatocellular-cholangiocarcinoma after resection. PLoS One.

[20] Sun HC, Tang ZY, Wang L, Qin LX, Ma ZC, Ye QH, Zhang BH, Qian YB, Wu ZQ, Fan J (2006). Postoperative interferon alpha treatment postponed recurrence and improved overall survival in patients after curative resection of HBV-related hepatocellular carcinoma: a randomized clinical trial. J Cancer Res Clin Oncol.

[21] European Association for the Study of the Liver (2018). Electronic address eee, European Association for the Study of the L: EASL clinical practice guidelines: management of hepatocellular carcinoma. J Hepatol.

[22] Rubin DB, Thomas N (1996). Matching using estimated propensity scores: relating theory to practice. Biometrics.

[23] Kim DH, Pieper CF, Ahmed A, Colon-Emeric CS (2016). Use and interpretation of propensity scores in aging research: a guide for clinical researchers. J Am Geriatr Soc.

[24] Garrido MM, Kelley AS, Paris J, Roza K, Meier DE, Morrison RS, Aldridge MD (2014). Methods for constructing and assessing propensity scores. Health Serv Res.

[25] Bruix J, Takayama T, Mazzaferro V, Chau GY, Yang J, Kudo M, Cai J, Poon RT, Han KH, Tak WY (2015). Adjuvant sorafenib for hepatocellular carcinoma after resection or ablation (STORM): a phase 3, randomised, double-blind, placebo-controlled trial. Lancet Oncol.

[26] Lee JH, Lee JH, Lim YS, Yeon JE, Song TJ, Yu SJ, Gwak GY, Kim KM, Kim YJ, Lee JW (2015). Adjuvant immunotherapy with autologous cytokine-induced killer cells for hepatocellular carcinoma. Gastroenterology.

[27] Ren ZG, Lin ZY, Xia JL, Ye SL, Ma ZC, Ye QH, Qin LX, Wu ZQ, Fan J, Tang ZY (2004). Postoperative adjuvant arterial chemoembolization improves survival of hepatocellular carcinoma patients with risk factors for residual tumor: a retrospective control study. World J Gastroenterol.

[28] Chen X, Zhang B, Yin X, Ren Z, Qiu S, Zhou J (2013). Lipiodolized transarterial chemoembolization in hepatocellular carcinoma patients after curative resection. J Cancer Res Clin Oncol.

[29] Li J, Wang Q, Lei Z, Wu D, Si A, Wang K, Wan X, Wang Y, Yan Z, Xia Y (2015). Adjuvant Transarterial chemoembolization following liver resection for intrahepatic Cholangiocarcinoma based on survival risk stratification. Oncologist.

[30] Kassahun WT, Hauss J (2008). Management of combined hepatocellular and cholangiocarcinoma. Int J Clin Pract.

[31] Kim JH, Yoon HK, Ko GY, Gwon DI, Jang CS, Song HY, Shin JH, Sung KB (2010). Nonresectable combined hepatocellular carcinoma and cholangiocarcinoma: analysis of the response and prognostic factors after transcatheter arterial chemoembolization. Radiology.

