# Supplementary material for: Adjuvant Transarterial chemoembolization does not influence recurrence-free or overall survival in patients with combined hepatocellular carcinoma and Cholangiocarcinoma after curative resection: a propensity score matching analysis
Source: BMC Cancer. 2020 Jul 10;20:642. doi: 10.1186/s12885-020-07138-z (PMC7350756; doi:10.1186/s12885-020-07138-z)
Supplement: Supplementary file 1 — Additional file 1. [file 12885_2020_7138_MOESM1_ESM.docx]

**Supplemental Materials**

**Patient Selection**

The inclusion criteria were as follows: (1) age, 18-75 years; (2) first combined hepatocellular carcinoma and intrahepatic cholangiocarcinoma after curative hepatectomy; (3) Child-Pugh class A; (4) adequate liver function (bilirubin < 3 mg/dL; alanine aminotransferase and aspartate aminotransferase < 5 times the upper limit of normal; alkaline phosphatase < 4 times upper limit of normal; prothrombin time < 6s above control); (5) adequate renal function (serum creatinine concentration < 1.5 times the upper limit of the normal range); (6) adequate hematologic function (hemoglobin concentration > 85 g/L, platelet count > 60 ×10^9^/L); (7) an Eastern Cooperative Oncology Group performance status score of 0.

The exclusion criteria were: (1) history of a secondary malignancy; (2) history of hepatic encephalopathy, ascites refractory to diuretics; (3) history of esophageal or gastric variceal bleeding; (4) previous or concomitant systemic therapy; (5) concomitant HIV infection (1).

Curative resection was defined as: (a) complete resection of tumor and with the tumor-free margin by histological examination; (b) no tumor thrombosis was existed in the portal vein including main trunk or two branches, hepatic veins, or bile ducts; (c) the number of tumor nodules did not exceed three; (d) no residual tumor in liver remnants on either contrast-enhanced computer tomography (CT) or magnetic resonance imaging (MRI).

**Variables and outcomes**

The data was prospectively collected and retrospectively reviewed. The database contained patient clinical characteristics, operative variables and follow-up information. All data were entered before the patients were discharged and after each follow-up visit in our outpatient department. The data were entered using “Epidata”. In our study, tumor satellite nodules were defined as those tumors presenting within 2 cm of a main tumor nodule and their sizes were less than 2cm in diameter. Multiple tumors were defined as two or more nodules of any size but more than 2cm from each other in distance.

**Follow-up**

Patients were followed up in our center every 3 months until death or dropout from the follow-up program. Abdominal ultrasound, liver function test, serum alpha-fetoprotein (AFP), carcinoembryonic antigen (CEA) and carbohydrate antigen 19-9 (CA19-9) levels were examined every 3 months, and abdominal MRI scans were performed every 6 months, respectively. Recurrence was diagnosed on the imaging findings from MRI or CT scans and increased serum AFP and CA19-9 levels. Chest CT and bone scintigraphy were used to assess extrahepatic recurrence. Regarding on the type of recurrence and liver function reserve, patients were treated with different treatments which comprised repeated resection, RFA, TACE, percutaneous ethanol injection (PEI), and chemotherapy for patients with extrahepatic metastatic disease.

**Reference**

1. Peng Z, Chen S, Xiao H, Wang Y, Li J, Mei J, Chen Z, Zhou Q, Feng S, Chen M, Qian G, Peng S, Kuang M. Microvascular Invasion as a Predictor of Response to Treatment with Sorafenib and Transarterial Chemoembolization for Recurrent Intermediate-Stage Hepatocellular Carcinoma. Radiology 2019;292(1):237-247. doi: 10.1148/radiol.2019181818
